# Role of Butyrate, a Gut Microbiota Derived Metabolite, in Cardiovascular Diseases: A comprehensive narrative review

**DOI:** 10.3389/fphar.2021.837509

**Published:** 2022-02-02

**Authors:** Parichehr Amiri, Seyed Ahmad Hosseini, Samad Ghaffari, Helda Tutunchi, Shamsi Ghaffari, Erfan Mosharkesh, Samira Asghari, Neda Roshanravan

**Affiliations:** ^1^ Student Research Committee, Ahvaz Jundishapur University of Medical Sciences, Ahvaz, Iran; ^2^ Nutrition and Metabolic Diseases Research Center, Clinical Research Institute, Ahvaz Jundishapur University of Medical Sciences, Ahvaz, Iran; ^3^ Department of Nutrition, School of Allied Medical Sciences, Ahvaz Jundishapur University of Medical Sciences, Ahvaz, Iran; ^4^ Cardiovascular Research Center, Tabriz University of Medical Sciences, Tabriz, Iran; ^5^ Endocrine Research Center, Tabriz University of Medical Sciences, Tabriz, Iran; ^6^ Faculty of Veterinary Medicine, University of Tabriz, Tabriz, Iran; ^7^ Stem Cell and Regenerative Medicine Institute, Tabriz University of Medical Sciences, Tabriz, Iran

**Keywords:** gut microbiota, butyrate, cardiovascular diseases, epigenetic modulation, antioxidant

## Abstract

Cardiovascular diseases (CVD) are major causes of death worldwide. Recently, new roles for intestinal microbiota in pathology and treatment of CVD have been proposed. Butyrate, a bacterial metabolite, is synthesized in the gut and performs most of its functions in there. However, researchers have discovered that butyrate could enter to portal vein and interact with various organs. Butyrate exhibits a broad range of pharmacological activities, including microbiome modulator, anti-inflammatory, anti-obesity, metabolic pathways regulator, anti-angiogenesis, and antioxidant. In this article we review evidence supporting a potentially therapeutic role for butyrate in CVD and the mechanisms and pathways involved in the cardio-protective effects of butyrate from the gut and circulation to the nervous system. In summary, although butyrate exhibits a wide variety of biological activities in different pathways including energy homeostasis, glucose and lipid metabolism, inflammation, oxidative stress, neural signaling, and epigenetic modulation in experimental settings, it remains unclear whether these findings are clinically relevant and whether the molecular pathways are activated by butyrate in humans.

## Introduction

Cardiovascular diseases (CVD) are disorders that affect the heart and blood vessels mainly including heart failure (HF), stroke, atherosclerosis, and hypertension (Ahmad et al., 2019). CVD are at the top of the life-threatening ailments list globally (Hulten et al., 2017). Based on the report of the World Health Organization (WHO), 17.9 million deaths were attributed to CVD in 2019, which accounted for 32% of all deaths (Roth et al., 2020).

Despite advances in primary prevention, CVD prevalence has risen in recent years (Tsivgoulis et al., 2018). The proven conditions that increase the risk of CVD including hypertension, dyslipidemia, obesity, and insulin resistance (IR) are concomitantly increased with CVD (Roth et al., 2020). In order to improve CVD prevention and treatment, it is essential to investigate unknown parts of the pathophysiology as well as to identify novel agents affecting risk factors (Singh et al., 2016; Warmbrunn et al., 2020). Dysbiosis of gut microbiota is one of the newest factors which is known to be involved in the development of CVD (Lau et al., 2017). Dysbiosis is characterized as alterations in microbial composition and their metabolites (Serino et al., 2014). An increasing body of evidence in CVD indicates alterations in microbial composition and their metabolites that may play a role in the pathogenesis and progression of these diseases (Brown and Hazen, 2015; Witkowski et al., 2020). The gut microbiota produces a wide variety of metabolites as a result of the anaerobic fermentation of undigested foods (Warmbrunn et al., 2020). Short-chain fatty acids (SCFAs) including acetate, propionate and butyrate, are main metabolites that may provide important protection.

Butyrate is a four-carbon SCFA, mainly known as a fuel for colonocytes. In addition to dietary fibers especially resistant starch, which is an indirect source of butyrate, some types of cheese, butter, and milk also contain small amounts of butyrate. Researches showed that butyrate can absorb into the portal vein and interact with the host body’s important processes like glucose homeostasis, lipid metabolism and gut inflammation. Several *in vitro* and *in vivo* studies have demonstrated that butyrate exerts anti-inflammatory, anti-oxidant, anti-obesity and metabolic regulation effects. The aim of the present study was to comprehensively review the therapeutic efficacy of butyrate in CVD as well as the mechanisms of action of butyrate on CVD risk factors.

## Butyrate Producing Bacteria and Alterations in CVD

**FIGURE 1:**
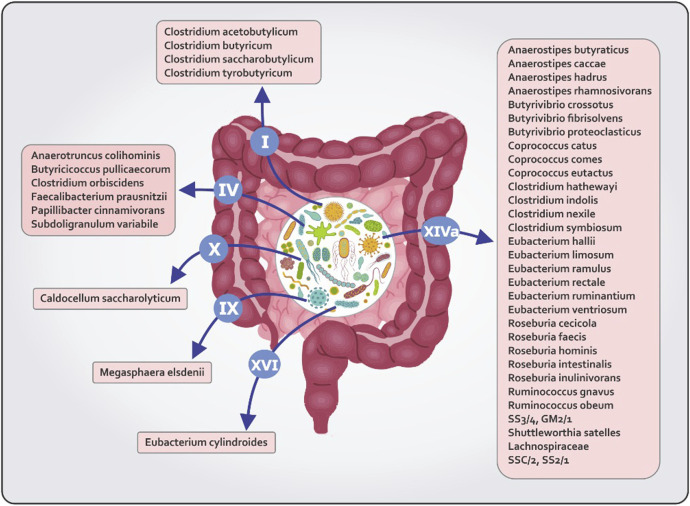
All butyrate producing bacteria in different clostridial clusters.

Gut microbiota is made up trillions of microorganisms including bacteria, viruses, fungi and protozoa (Ley et al., 2006). A variety of dietary substrates are used by these microbes to produce a range of metabolites, some of which are beneficial to the host (Scott et al., 2013). The production of butyrate is widespread among diverse phyla of human colon (Louis and Flint, 2009). Firmicutes and *Bacteroides* are the main butyrate producing phyla (Chen et al., 2020). The identification of butyrate producing spices has been done using sequence based detection algorithms such as metagenomics and 16S ribosomal RNA sequencing (Louis and Flint, 2009). All butyrate-producing bacteria are shown in Figure 1. The most prominent butyrogenic bacteria groups are *Faecalibacterium prausnitzii*, *Butyrivibriocrossotus* and *Roseburia intestinalis* which several studies have reported the depletion of these bacteria in atherosclerosis (Chen et al., 2020). Kasahara et al. demonstrated that the abundance of Roseburia intestinalis is negatively correlated with the size of atherosclerotic lesions in the mouse model of atherosclerosis (Kasahara et al., 2018). In that study, when Roseburia intestinalis was taken along with a high-fiber diet, aortic atherosclerotic plaques were reduced in size. This study suggests that the butyrate, a microbial metabolite, mediates these effects (Kasahara et al., 2018). A research conducted by zeng et al. illustrated that bacteria with capacity of butyrate production, Lachnospiraceae and Ruminococcaceae, were depleted in individuals at a high risk of stroke. Fecal butyrate concentrations also were low in these people (Zeng et al., 2019). Additionally, metagenomes analysis of individuals with symptomatic carotid atherosclerosis showed that gene expression of butyrate-synthesizing enzyme (butyrate-ace- toacetate CoA-transferase) were inversely correlated with C reactive protein (CRP) levels (Karlsson et al., 2012).

Several studies have reported that patients with HF have a decrease in butyrate-producing bacteria, especially, Lachnospiracea and Ruminococcacea families (Trøseid, 2020). Remarkably, reduction of the butyrate-producing Eubacterium Halli and Lachnospiracea is correlated with increased inflammation, severity of disease, heart damage and mortality (Kummen et al., 2018). Moreover, there is evidence that dysbiosis is related to low butyrate production in different HF cohorts (Trøseid, 2020). Likewise, animal and human studies reported decrease in bacteria with capacity of butyrate production in hypertension (Mell et al., 2015; Yang et al., 2015; Gomez-Arango et al., 2016; Yan et al., 2017). According to these studies, amount of butyrate producer bacteria is negatively associated with blood pressure (BP) (Santisteban et al., 2017).

Overall, the evidence suggests that decrease in butyrate-producing bacteria abundance, down regulation of genes involved in butyrate synthesis, and low butyrate levels are associated with sensitivity of developing CVD.

## Mechanisms of Action of Butyrate in CVD and CVD Risk Factors

### Major Mechanisms

The key aspects of butyrate mechanism of action can be listed as follows. First, butyrate has reported to be an epigenetic modifier by acting as a histone deacetylases (HDACs) inhibitor (Chang et al., 2014). HDACs are chromatin-modifying enzymes that alter genes transcription accessibility by removing acetylate from histones and non-histone proteins (Shakespear et al., 2011). The abnormal regulation of gene expression underlies many human metabolic disorders such as CVD. By inhibiting HDACs, butyrate causes hyper-acetylation of transcription factors and regulates gene expression patterns (Cleophas et al., 2019).Second, butyrate influences cellular responses by binding and activating specific receptors named free fatty acid receptor 2 (FFAR2) and FFAR3 (Previously these receptors were called G protein-coupled receptors (GPRs), GRP 43 and 41 respectively) (Brown et al., 2003).

Butyrate can contribute in widespread cardiovascular-related functions through inducing intracellular signaling pathways by interacting with FFARs on target cells or causing epigenetic changes by inhibiting HDAC.

### Butyrate as a PPARs Agonist

Butyrate is known to be a pleiotropic molecule that as well as binding to FFARs, also has the ability to bind peroxisome proliferator-activated receptors (PPARs) (Are et al., 2008; Alex et al., 2013; Korecka et al., 2013; Mattace Raso et al., 2013; Den Besten et al., 2015; Chitrala et al., 2017). PPARs are a family of ligand-activated transcription factors that recognized to have a significant impact on metabolism related pathways. Due to several regulatory roles in metabolic function and energy hemostasis, they are now one of the most often proposed therapeutic targets for metabolic disorders (Oh et al., 2019). Butyrate could induce adipogenesis by activating PPARγ, which is supported by several studies (Toscani et al., 1990; Li et al., 2014; Yan and Ajuwon, 2015). Adipogenesis is associated with reduced inflammatory and oxidative molecules production in adipose tissue, organs lipotoxicity and IR (Hafidi et al., 2019). In diet-induced obese Apo E−/− mice, oral administration of sodium butyrate (SB) (10 ml/kg diet) increased vascular endothelial growth factor (VEGF) mediated vascularization via upregulating PPARγ contributing to improvement of angiogenesis, inflammation and insulin sensitivity (Aguilar et al., 2018). Anti-angiogenic properties of butyrate have also been proposed both *in vitro* and *in vivo*, which is associated with upregulation of anti-angiogenic VEGF (Ciura and Jagodziński, 2010). Moreover, upregulation of PPARγ by butyrate modulates nuclear factor-κB (NF-κB) pathway, resulting in improvements in insulin signaling and inflammation (Aguilar et al., 2018).

On the other hand, SB may activate AMP kinase (AMPK), which result in upregulation of PPARγ coactivator (PGC)-1α, PPARα and γ (Mattace Raso et al., 2013; Hong et al., 2016). Activation of PPARs induces mitochondria biogenesis through upregulating uncoupling proteins (UCPs) and increases fatty acid oxidation. It is speculated that this is an adiponectin-mediated pathway and recruitment of this pathway starts with upregulation of adiponectin receptors as a result of butyrate supplementation (Hong et al., 2016).

## The Effects of Butyrate on CVD

### Butyrate and Atherosclerosis

An increasing number of studies have found that butyrate can exert protective effects in atherosclerosis. Aguilar et al. first found that supplementation of diet with 1% butyrate could reduce atherosclerotic lesions in ApoE knockout mice by decreasing adhesion molecules production and reducing migration of macrophage to the lesion site (Aguilar et al., 2018). Further *in vivo* research has shown that incubation of macrophage and endothelial cells with butyrate (0.5 mM) for 2 h can increase interleukin-10 (IL-10) production meanwhile can decrease pro-inflammatory cytokines including tumor necrosis factor α (TNFα), IL-1β and IL-6 mainly through suppressing NF-κB pathway. The uptake of oxidized-low density lipoprotein (oxLDL) was also decreased by butyrate treated cells (Aguilar et al., 2018). In Kasahara et al. study, the mice group fed with a 6% tributyrin (TB)-supplemented diet had lower lipid deposition and macrophage accumulation in the lesion. It was also reported that butyrate resulted in improvement of gut permeability (Kasahara et al., 2018).

In high-fat diet-fed ApoE^−/−^ mice, butyrate administration (200 and 400 mg/kg) influenced the microbial composition of the gut and improved diversity in favor of increasing Firmicutes specially Bacteroidetes (Du et al., 2020). Additionally, butyrate ameliorated atherosclerosis by downregulating genes involved in lipid metabolism including acyl-CoA thioesterase1 (Acot1), Acot2, Perilipin2 (Plin2), Plin5, Cytochrome4a (10,14 and 31 isoforms) (Du et al., 2020). Results of this study showed that, cytochrome P450 7A1 (CYP7A1) is upregulated in butyrate treated mice and negatively correlated with atherosclerotic lesions (Du et al., 2020). CYP7A1 controls bile acid biosynthesis and helps for the elimination of cholesterol in the liver. This evidence suggests a potential role of butyrate in bile acid metabolism (Li et al., 2013). In the same study, *in vivo*, *ex vivo* and *in vitro* investigations illustrated that butyrate induces ATP-binding cassette subfamily A member 1 (ABCA1) activity in both hepatocytes and macrophages via transcription factor specific protein 1 (Sp1), which causes reduced total cholesterol (TC) and also cholesterol deposition in plaque (Du et al., 2020). ABCA1 is a crucial transporter that contributes to cholesterol efflux toward the biosynthesis of high-density lipoprotein-cholesterol (HDL-C) (Sahoo et al., 2004). Previous studies have shown ABCA1 constrains the formation of foam cells, which are involved in the development of atherosclerosis (Chawla et al., 2001; Joyce et al., 2002).

Inflammation and oxidative stress are well-known in the pathogenesis of atherosclerosis (Ma et al., 2016). In Wang et al. study, butyrate (100 and 200 μM) showed anti-inflammatory and antioxidants effects on TNF-α induced human umbilical vein endothelial cells (HUVECs) by decreasing adhesion molecules (vascular cell adhesion molecule-1 (VCAM-1) and E-selectin) and subsequent THP-1 monocytes attachment, reducing pro-inflammatory cytokines including monocyte chemoattractant protein-1(MCP-1) and IL-8, attenuating oxidants ROS and 4-hydroxy nonenal (4-HNE), improving the protective function of kruple factor 2 (KLF2) via the extracellular-signal-regulated kinase 5 (ERK5) pathway (Wang et al., 2020).

Taken together, these findings indicate that athero-protective effects of butyrate are accompanied by regulating the expression of genes related to lipid and glucose metabolism, improving gut microbiota diversity, suppressing a wide range of inflammatory and oxidative processes, rescuing protective KLF2, and enhancing vascular health. Summary of the studies about the effects of butyrate on atherosclerosis are shown in Table 1.

**TABLE 1:** Summary of the studies about the effects of butyrate on atherosclerosis.

Reference	Type of study	Butyrate dose	Model	Result
Aguilar et al. (2018)	*In vivo*	1%wt/wt butyrate in diet for 10 weeks	HFD fed ApoE knockout mice	decreased atherosclerosis lesions of aorta
				decreased CCL2, VCAM1
				increased MMP2 and9
				decreased migration of macrophage
				increased collagen depositions and plaque stability
	*In vitro*	0.5 mM butyrate for 2 h	Macrophage	decreased ox-LDL uptake, CD36, VCAM1, CCL2, TNFα, IL1β and IL6
			endothelial cells	increased IL10 levels
				inhibited NF-kB activity
Kasahara et al. (2018)	*In vivo*	6% wt/wt tributyrin in diet for 14 weeks	ApoE knockout mice	inhibited the development of atherosclerosis, lipid deposition and macrophage accumulation in the plaque
				reduced gut permeability
Du et al. (2020)	*In vivo*	SB 200 and 400 mg/kg/day for 16 weeks	HFD fed ApoE knockout mice	improved the gut microbial diversity
				increased the abundance of Firmicutes
				decreased cholesterol deposition
				decreased atherosclerotic lesions of aortae
				decreased TC
				increased the ABCA1 level in liver
	*In vitro*	2 and 5 mM butyrate for 24 h	Murine RAW 264.7 macrophages	increased ABCA1 protein level
			ABCA1p-Luc HepG2 cells	increased the cholesterol efflux in RAW 264.7 macrophages in a dose-dependent manner
			Primary peritoneal macrophages	—
Wang et al. (2020)	*In vitro*	100 and 200 μM butyrate for 24 h	TNF-α induced HUVECs cells	decreased VCAM-1 and E-selectin
				reduced oxidative stress by reducing the levels of ROS and 4-HNE
				decreased MCP-1 and IL-8
				improved protective factor KLF2,via the ERK5 pathway

Abbreviations: HFD, high fat diet; APO E, apolipoprotein E; CCL2, C–C motif chemokine ligand 2; VCAM1, vascular adhesion molecule-1; MMP, matrix metalloproteinases; ox-LDL, oxidized-low density lipoprotein; TNFα, Tumor necrosis factorα; IL, interleukin, NF-kB, Nuclear factor kappa B; SB, sodium butyrate; Hep G2, hepatocye G2; TC, total cholesterol; ABCA1, ATP Binding Cassette Subfamily A Member 1; HUVECs, human umbilical vein endothelial cells; ROS, reactive oxygen species; 4-HNE, 4-Hydroxynonenal; MCP1, monocyte chemoattractant protein-1; KLF2, Kruppel Like Factor 2; ERK5, Extracellular-signal-regulated kinase 5.

### Butyrate and Heart Failure

HF is a condition in which the heart’s ability to fill or evacuate blood is compromised (Jin et al., 2020). HF can be caused by any problem that affects the anatomical and/or functional integrity of the heart, such as valve, coronary, or myocardial disease (Luedde et al., 2017).

In a recent study by Mollar et al., butyrate was negatively associated with area under the concentration curve (AUC-H2) in patients with HF, implying that butyrate is lower in patients with higher exhaled hydrogen test (Mollar et al., 2021). Although hydrogen breath tests are not the gold standard for diagnosing small intestinal bacterial overgrowth (SIBO), the results of the aforementioned study suggest that dysbiosis followed by reduced levels of butyrate could play a role in the pathology of HF (Ghoshal, 2011; Mollar et al., 2021). The findings of both *in vivo* and *in vitro* experiments show that butyrate exerts histological cardio-protective effects. Badejogbin et al. examined the impact of SB (200 mg/kg) on heart tissue damage in rats fed either chow or high fat diet (HFD) (Badejogbin et al., 2019). In that research, butyrate significantly ameliorated HFD induced cardio-metabolic abnormalities including hyperlipidemia and glucose dysmetabolism as well as elevated plasma malondialdehyde, corticosterone, and lactate dehydrogenase (Badejogbin et al., 2019). Furthermore, the histological study revealed that butyrate improved cardiac tissue infarction, infiltration, and fibrosis. Butyrate has been shown to protect cardiac tissue architecture and integrity by lowering uric acid (plasma and cardiac tissue) and increasing glutathione antioxidant defenses (Badejogbin et al., 2019).

The incubation of endothelin-1 (ET1) induced cardiomyocytes derived from neonatal rats with butyrate (1–4 mM) inhibited hypertrophic growth of cardiomyocytes by epigenetic gene expression alterations (Umei, 2020). In the same study, transcriptome analysis demonstrated that FFARs didn’t have an expression in cardiomyocytes therefore protective anti-hypertrophic action is related to HDAC inhibitory role of butyrate.

In a study conducted by Jiang et al., intraperitoneal butyrate administration (7.5 mg/kg) in rats with myocardial infarction reduced the region of infarction and increased cardiac function by enhancing M2 macrophage polarization, downregulating the expression of inflammatory response-related genes, and suppressing sympathetic nerve remodeling (Jiang et al., 2020). Moreover, SCFAs have been found to influence sympathetic neurons, with the effects mostly relying on the vagus nerve. It seems gut and brain are interconnected mostly via the vagus nerve (Yu et al., 2020).

A growing body of evidence suggests that in addition to butyrate’s action on anatomical features of the heart, butyrate may also act through gut-brain neurological processes especially vagal afferent pathway (Li et al., 2018; Onyszkiewicz et al., 2019; Muller et al., 2020; Yu et al., 2020). In this context, li et al. research showed that SB supplementation (5% w/w) improved energy metabolism in HFD fed rats, through the gut-brain neural circuit and these effects diminished after vagotomy (Li et al., 2018). Yu et al. investigated the effect of SB (200 mmol/L) on reperfusion injury in rats underwent vagotomy + myocardial ischemia/reperfusion (I/R) injury or I/R injury alone (Yu et al., 2021). According to previous studies, reperfusion can reduce cardiac function and is associated with an increased risk of HF (Goel et al., 2013). Butyrate significantly reduced infarct size and myocardial damage indicators (plasma lactate dehydrogenase (LDH), creatine kinase (CK), and CK-MB levels). Likewise, butyrate treated rats showed a decrease in I/R-induced oxidative stress, inflammation, and apoptosis. Nonetheless, these effects reversed with a vagotomy (Yu et al., 2021). It is speculated that butyrate improves myocardial I/R injury through the gut-brain neural circuit, and this cardio-protective effect is probably mediated by suppressing sympathetic nervous system. Summary of the studies about the effects of butyrate on HF are shown in Table 2.

**TABLE 2:** Summary of the studies about the effects of Butyrate on heart failure.

Reference	Type of study	Butyrate dose	Model	Result
Badejogbin et al. (2019)	*In vivo*	200 mg/kg/day butyrate in diet for 6 weeks	HFD fed wistar rats	ameliorated glucose dysmetabolism
				decreased TG, TC, corticosterone, MDA, plasma and cardiac UA, and LDH
				Increased glutathione
				Reduced cellular infarction, infiltration, and fibrosis
Aguilar et al. (2018)	*In vitro*	1, 2, 4 mM butyrate for 2 h	endothelin-1 (ET1) induced neonatal cardiomyocytes	inhibited hypertrophic growth of cardiomyocytes
Jiang et al. (2020)	*In vivo*	7.5 mg/kg/day butyrate intraperitoneally injected for 3 or 7 days post MI	Sprague-Dawley rats MI model	increased expression of M2 macrophage markers
				downregulated expression of inflammatory response-related genes
				suppressed sympathetic nerve remodeling
				inhibited myocardial hypertrophy
Yu et al. (2021)	*In vivo*	200 mmol/L SB in drinking water for 4 weeks	Sprague-Dawley rats myocardial ischemia/reperfusion (I/R) injury model	decreased infarct size
				decreased myocardial damage indicators (CK, CK-MB and LDH)
				decreased inflammation, oxidative stress, and apoptosis
				suppressed sympathetic nervous system
				protective effects were diminished by vagotomy

Abbreviations: HFD, high fat diet; TG, Triglycerides; TC, total cholesterol; MDA, malondialdehyde; UA, uric acid; LDH, lactate dehydrogenase; MI, myocardial infarction; SB, sodium butyrate; CK, creatine kinase; CK-MB, creatine kinase myocardial isoenzyme.

### Butyrate, Hypertension, and Vascular Health

It is hypothesized that butyrate’s modulatory effects on BP occur through its interaction with the circulatory system**.** In Sprague-Dawley rats, intramedullary butyrate treatment decreased angiotensin II (Ang II) ‐induced mean arterial pressure via suppression of (pro) renin receptor (PRR) and its subsequent intrarenal renin-angiotensin system (Wang et al., 2017).

Zhang et al. showed that SB administration (1 g/kg/d) inhibited the activation of the cyclooxygenase-2 (COX-2)/prostaglandin E2 (PGE2) pathway in a HDAC5/HDAC6-dependent manner, contributing to reducing Ang II-induced heart hypertrophy, mean arterial pressure and inflammation (Zhang et al., 2019). Robles-Vera et al. investigated the cardiovascular effects of butyrate (0.5 mg kg day) in spontaneously hypertensive rats (SHR) and control Wistar Kyoto (WKY) rats (Robles‐Vera et al., 2020). Butyrate decreased both systolic and diastolic BP and returned T-helper 17 (Th17)/regulatory T cells (Treg) balance in the SHR to WKY rat levels. These effects are mediated by lowering endotoxemia and increasing Treg cells in the vasculature (Robles‐Vera et al., 2020). Butyrate in the blood circulation stimulates FFAR3 which is found in veins and contributes to vascular tone. FFAR3 is a hypotensive protein, dilates resistance vessels in an endothelium-dependent manner (Natarajan et al., 2016). Furthermore, FFAR2 and FFAR3 have expression in nerves and evidence revealed their expression is higher in WKY than in SHR rats, therefore, intra brain butyrate administration in WKY had a higher drop in BP than SHR (Toral et al., 2019; Yang et al., 2019). Onyszkiewicz et al. showed that butyrate administration (1.4, 2.8, and 5.8 mmol/kg, intracolonic) caused dose-dependent BP reduction in rats fed a standard diet. It seems these hypotensive effects are mediated via afferent colonic vagus nerve vasorelaxation signaling and FFAR2/3 (Onyszkiewicz et al., 2019).

Nutting and Mortesen studies on arties showed that butyrate causes endothelial-dependent vasodilation in the arteries by increasing cyclic AMP (cAMP) levels (Mortensen et al., 1990; Nutting et al., 1991). In Morikawa et al. study, butyrate administration (1 mM) enhanced nitric oxide (NO) production in interferon treated vascular endothelial cells via increasing expression of inducible NO synthase (iNOS) (Morikawa et al., 2004). It is well known that NO stimulates vasodilation, reduces inflammation, and lowers BP (Napoli et al., 2010). Summary of the studies about the effects of butyrate on hypertension and vascular health are shown in Table 3.

**TABLE 3:** Summary of the studies about the effects of butyrate on hypertension.

Reference	Type of study	Butyrate dose	Model	Result
Wang et al. (2017)	*In vivo*	1 g/kg/day SB for 14 days	Ang II‐infused sprague-Dawley rats model of HTN	decreased Ang II-induced mean arterial pressure
				decreased gene expression of TNFα and IL6
Zhang et al. (2019)	*In vivo*	1 g/kg/day SB for 2 weeks	Ang II‐infused Sprague Dawley rats model of HTN	decreased Ang II-induced mean arterial pressure
				decreased gene expression of IL‐1β, Nlrp3, and MCP‐1 in cardiac tissue
	*In vitro*	2 mmol/L SB	cardiomyocytes H9C2 cells	inhibited cardiac hypertrophy by inhibiting COX2/PGE2 pathway
	Onyszkiewicz et al. (2019)	*In vivo*	1.4, 2.8, and 5.8 mmol/kg/day, Intracolonic (IC) or intravenously (IV) butyric acid for 2 days	Wistar rats	IC: increased concentration of butyric acid in the colon, portal and systemic blood, decreased BP and heart rate
IV: decreased BP didn’t changed heart rate				
hypotensive effect was depended on vagus nerve signaling and FFAR2/3 receptors				
*Ex vivo*		5 μM up to 1 mM butyric acid	mesenteric arteries (MA)	butyric acid dilated MA and GMA
			gracilis muscle arteries (GMA)	effective dose was 50 μM up to 1 mM
Robles‐Vera et al. (2020)		*In vivo*	0.5 mg/kg/day SB for 13 weeks	WKY and SHR Rats	prevented increase in systolic and diastolic BP
	prevented increase in Firmicutes/Bacteroidetes (F/B) ratio			
	increased Th17/Treg balance			
	decreased endotoxemia			

Abbreviations: SB, sodium butyrate; Ang II, Angiotensin II; TNFα, Tumor necrosis factorα; IL, interlukin; HTN, hypertension Nlrp3; MCP‐1, monocyte chemoattractant protein; COX2, cyclooxygenase‐2; PGE2, prostaglandin E2; IC, intracolonic; IV, intravenously; BP, blood pressure; FFAR, free fatty acid receptor; MA, mesenteric arteries; GMA, gracilis muscle arteries; WKY, wistar Kyoto rat; SHR, spontaneously hypertensive rat; Th, T helper; Treg, T regulatory.

## Butyrate and CVD Risk Factors

### Butyrate and Obesity

Several scientific studies confirm obesity as an independent risk factor for CVD, as well as one of the factors increasing the risk of diseases associated with CVD such as dyslipidemia, IR, hypertension, and atherosclerosis (Cercato and Fonseca, 2019). In a recent review by Bridgeman et al., out of 14 studies that examined the effects of butyrate on obesity in HFD fed animals, butyrate significantly was reduced weight gain in 10 studies (Bridgeman et al., 2020). Butyrate exerts its anti-obesity effects mainly through contributing in energy balance equation. First, it reduces calorie intake by reducing appetite and preventing food intake. Second, butyrate increases energy expenditure by affecting metabolic pathways. According to the previous studies, butyrate reduces appetite by increasing anorexic hormones like peptide YY (PYY), glucagon-like peptide 1 (GLP1) through activating FFARs (Lin et al., 2012; Steinert et al., 2017). In a study by li et al., acute and chronic oral butyrate administration (5% (w/w) SB) by attenuating hypothalamic neuronal signaling decreased food intake in HFD mice (Li et al., 2018). However, butyrate had no effect on food intake after vagotomy in mice, suggesting the gut-brain neural circuit involvement. Since it is shown that GLP-1 contributed in the satiety, it may affect the vagal nerve (Li et al., 2018). In addition, butyrate induces the secretion of leptin and adiponectin from adipocytes which are involved in appetite and food intake control (Hong et al., 2016; Yu et al., 2017; Hafidi et al., 2019).

Evidence shows that butyrate increases thermogenesis and promotes fat oxidation by activating brown adipose tissue (BAT), thereby enhancing energy expenditure in the body. Li and hong et al. studies reported that rats consuming butyrate in their diet increased expression of UCPs in BAT and skeletal muscles (Hong et al., 2016; Li et al., 2018). UCPs are mitochondrial proteins and involved in facilitating heat production (thermogenesis) (Ricquier and Bouillaud, 2000). Moreover, the level of tyrosine hydroxylase protein was elevated in BAT by butyrate, which is linked to sympathetic nervous system activity. Therefore, butyrate-induced appetite reduction and BAT activation may depend on gut-brain neural circuitry and vagal nerve signaling (Li et al., 2018).

However, there are no randomized clinical trials that confirm the anti-obesity effect of butyrate and still need to be studied. There is no doubt that weight management strategies can help to combat obesity-related diseases including CVDs (Ebbert et al., 2014). As a result, butyrate supplementation can be considered as an emerging anti-obesogenic agent in the prevention and treatment of obesity and cardio-metabolic disease.

### Butyrate and Dyslipidemia

Several studies have demonstrated that butyrate has beneficial effects on dyslipidemia and may be helpful for lowering the risk of CVD, particularly atherosclerosis. We will review the effects of butyrate on triglycerides and cholesterol. The effect of butyrate on triglyceride levels was inconsistent in different studies. Du and Hong et al. reported that butyrate supplementation did not change triglyceride levels (Hong et al., 2016; Du et al., 2020), where as in Li and khan studies butyrate significantly decreased triglyceride levels (Khan and Jena, 2016; Li et al., 2018). Evidence suggests that butyrate has effects on adipogenesis, lipogenesis, and lipolysis (Toscani et al., 1990; Lu et al., 2012; Li et al., 2014; Rumberger et al., 2014; Yan and Ajuwon, 2015; Yu et al., 2017). The positive effect of butyrate on adipogenesis has been reported to be more pronounced. As previously discussed, butyrate promotes adipogenesis by activating the PPAR pathway, thereby reducing circulating fatty acids and their accumulation in vital organs (Hafidi et al., 2019). Furthermore, in Aguilar et al. study, remodeling and proliferation markers such as matrix metalloproteinases (MMP2 and MMP9) and proliferating cell nuclear antigen (PCNA) increased following the increase in the expression of PPAR (Aguilar et al., 2018). MMPs and PCNA are required components for adipogenesis (Blaut and Clavel, 2007; Bauters et al., 2015). Hence, butyrate prevents metabolic disorders such as IR, dyslipidemia, and fatty liver by increasing adipogenesis.

The effect of butyrate on lipogenesis and lipolysis is contradictory and seems to be due to differences in the dose, duration of butyrate treatment, and cell phenotype in the *in vitro* studies. Therefore, since butyrate increases lipolysis in a number of studies and inhibits lipolysis in others, the definitive conclusion about the effect of butyrate on lipid metabolism in adipocytes requires further studies.

Butyrate supplementation has been shown to lower total serum cholesterol in a number of animal studies (Gao et al., 2009; Mattace Raso et al., 2013; Khan and Jena, 2016; Mollica et al., 2017; Zhao et al., 2017; Hu et al., 2018) Cholesterol in the body has two sources, dietary cholesterol and endogenous cholesterol (Kapourchali et al., 2016). According to studies, butyrate can affect both pathways of endogenous cholesterol biosynthesis and dietary cholesterol uptake. Butyrate treatment (200 and 400 mg kg) in HFD fed mice caused reduction in non-high-density lipoprotein cholesterol (non-HDL-C), low-density lipoprotein cholesterol (LDL-C), and total cholesterol (TC) (Du et al., 2020). It is noteworthy that regression analyze revealed that non-HDL-C, LDL-C and TC were positively correlated with percentage of aortic lesions, suggesting that butyrate by down-regulating fatty acid synthesis genes, modifies serum lipid levels and inhibits the progression of atherosclerosis (Du et al., 2020). Alvaro and Marcil reported butyrate decreased 3-hydroxy-3-methyl-glutaryl-coenzyme A (HMG-CoA) reductase gene expression and activity in Caco-2 cells (Marcil et al., 2003; Alvaro et al., 2008). The effect of butyrate on cholesterol biosynthesis is mainly through the downregulation of genes involved in cholesterol synthesis such as isopentenyl diphosphate isomerase, dimethylallyl/geranyl *trans*-transferase and farnesyl-diphosphatase farnesyltransferase. In addition, butyrate prevents intestinal absorption of cholesterol and lowers cholesterol levels. Chen et al. reported that butyrate downregulated Niemann-Pick C1-Like 1 which is involved in intestinal cholesterol uptake (Chen et al., 2018).

### Butyrate and Insulin Resistance

There is considerable evidence that both IR and its clinical manifestation, metabolic syndrome, are linked to CVDs (Abdul-Ghani et al., 2019). Numerous studies have shown butyrate can cause an increase in insulin sensitivity by increasing activity of insulin receptors through enhancing insulin receptor substrates. As a result of increased insulin signaling, glucose uptake by cells increases and hyperglycemia alleviates (Yan and Ajuwon, 2015). An increase in glucose transporters (GLUTs) including GLUT2 and GLUT4 after treatment by butyrate has been reported which is further support this hypothesis (Mollica et al., 2017). As mentioned earlier butyrate is also involved in improving insulin sensitivity by increasing adipogenesis (Hafidi et al., 2019). Furthermore, there is evidence indicating that butyrate is effective in reducing hyperglycemia by reducing the expression of gluconeogenesis related genes (Mihaylova et al., 2011; Khan and Jena, 2016). On the other hand, oxidative stress can cause IR with disrupting insulin signaling (Evans et al., 2005). SB administration has been found to reduce oxidative stress in a variety of tissues and cells (Sun et al., 2019). SB induces nuclear factor E2-related factor 2 (Nrf2) and increases expression of downstream antioxidant enzymes, thus contributes in the amelioration of oxidative stress and IR (Sun et al., 2019).

## Knowledge Gaps and Future Directions

Numerous *in vivo* and *in vitro* studies have been conducted exploring the direct and indirect cardiovascular protective capacities of butyrate. The existing body of research on butyrate efficacy suggests that butyrate health promoting effects are mainly due to its two main properties being HDAC inhibitor and activating FFARs. However, in most studies it is not clear exactly, these positive effects are related to which feature. In addition, recent studies have shown new ability such as binding to PPARs that require further study.

However, there is a paucity of clinical trials evaluating efficacy of butyrate supplementation in CVD prevention and treatment. Hence, the main knowledge gap is the lack of human clinical trials to investigate therapeutic benefits of butyrate in CVD. Certainly, large-scale clinical trials with detailed insights of the mechanisms involved are required to confirm the promising effects of butyrate in the management of CVD.

Based on evidence, small amounts of butyrate reaches systemic circulation due to high hepatic clearance (van der Beek et al., 2015). Consequently, placebo-controlled trials of butyrate supplements and butyrate generating bacteria are recommended to determine whether they are effective at increasing systemic butyrate levels. Moreover, Clinical trials are needed to examine the effects of butyrate on AMPK signaling pathway factors like adiponectin receptors, PGC-1α and UCPs which are contributed in energy hemostasis and lipid metabolism.

## Conclusion

**FIGURE 2:**
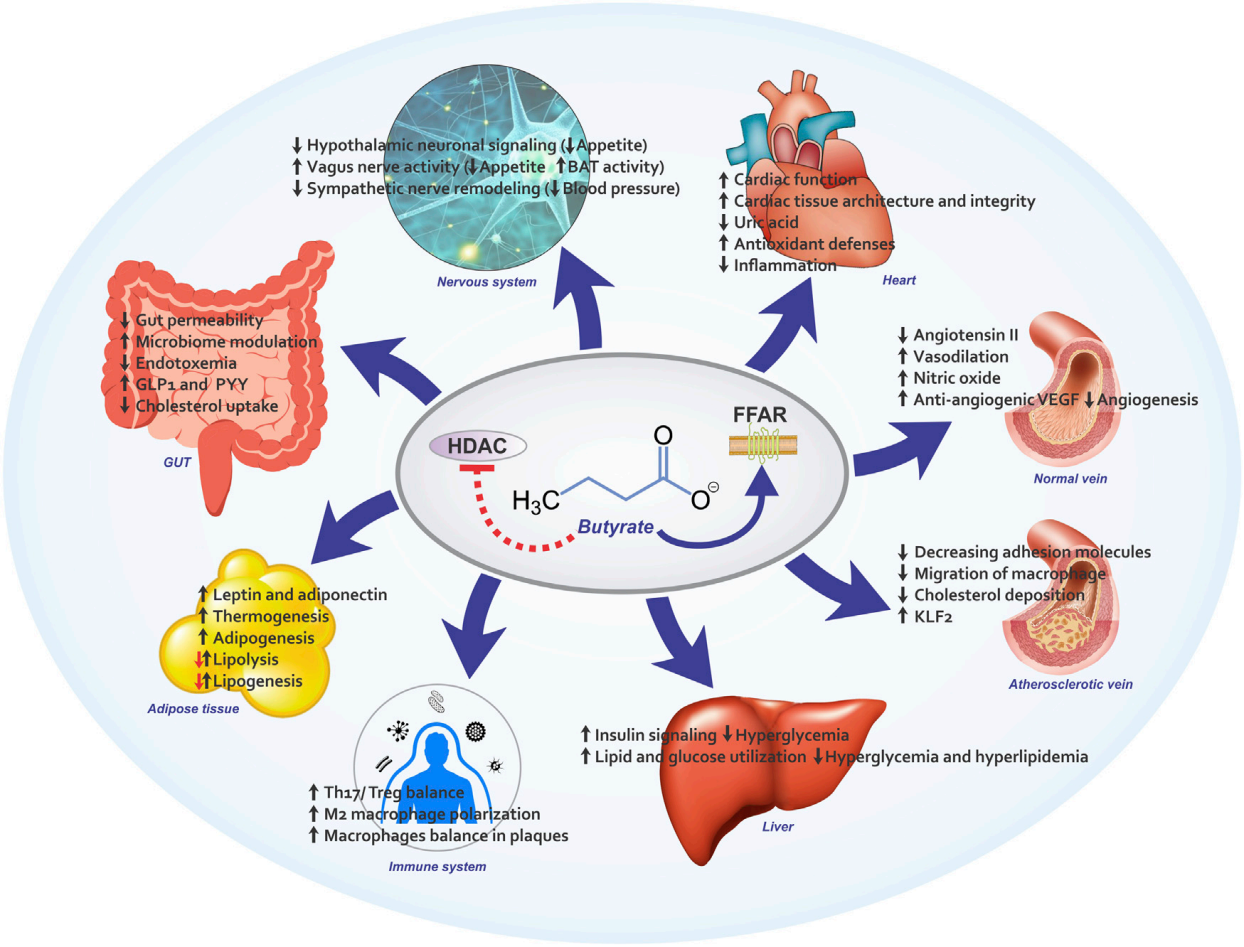
An overview of butyrate’s protective effects in CVD and CVD risk factors. FFAR: free fatty acid receptor, HDAC: Histone deacetylase, KLF2: Kruppel Like Factor 2,VEGF: vascular endothelial growth factor, GLP-1: glucagon-like peptide 1, PYY, peptide YY.

Butyrate has been shown favorable effects in the animal models of CVD as well as CVD-related risk factors such as obesity, dyslipidemia and IR. The beneficial results of butyrate go beyond the gut and it affects various organs as shown in Figure 2. Although butyrate exhibits a wide variety of biological activities in different pathways including energy homeostasis, glucose and lipid metabolism, inflammation, oxidative stress, neural signaling, and epigenetic modulation in experimental settings, it remains unclear whether these findings are clinically relevant and whether the molecular pathways are activated by butyrate in humans. Considering several factors that may contribute to cardio-protective activities of butyrate, further well-designed studies are needed to focus on these factors. Understanding exact mechanisms of butyrate in humans will facilitate the application of butyrate as a safe supplement in prevention and management of CVD.
